# Vital Statistics of Buffalo

**Published:** 1880-03

**Authors:** 


### VITAL STATISTICS OF BUFFALO.

The medical profession of this city is to be congratulated upon the zeal and industry which has recently been exhibited by its Board of Health. The gentlemen, lately elected to that position, have evidently entered upon their duties with a realization of the responsibilities attending it, and their efforts even thus far, in behalf of sanitary science, can not be too highly appreciated.

A "statement of mortality" is now sent weekly to every physician in the city, and this report is so much more complete than any formerly furnished, as to be deserving of special praise.

The vital statistics are here presented under different tabulated heads, in such a manner as to be very convenient for reference or special duty.

The general plan of the report is similar to that proposed by the National Board of Health, with some minor modifications suggested by our city physician, Dr. A. H. Briggs.

The causes of death are arranged in six classes, which are subdivided into forty-nine sections. The first of these classes includes the zymotic diseases and is composed of sixteen sections, representing as many different affections. These are also so arranged as to show the number of deaths from each disease in each of the different wards of the city—an advantage plainly evident in case of an epidemic of any kind.

The second class includes the "constitutional diseases;" to the third belong the "local diseases" of the system, etc., etc. Moreover, these are so tabulated as to show the approximate age of the deceased, together with the color, sex and social relation.

In other tables can be found the deaths reported in public institutions, and the births, marriages, and still-births during the week, while on the fourth page there is a summary of the daily meteorological observations, furnished for the same period from the office of the signal service.

We understand that efforts have been made by the Health Physicians in former years, to compel greater exactness in the recording of vital statistics, and then to report these in a convenient form to the profession, but unfortunately such proposals did not find favor with the other members of the Board of Health at that time.

But the present city physician has come into office full of enthusiasm for the work, his suggestions are duly weighed by intelligent men with whom he is associated, and as a result, we already see evidences of an interest in sanitary science, which has not formerly been manifested.

This whole subject of vital statistics is of such importance as to demand a more extended notice than can be given it at present. In the future we shall attempt to call attention to other reforms much needed; but at this time we only wish to commend the Board of Health for the action already taken, and assure them that all such efforts will meet the hearty approval of every intelligent physician in the community.

